# Antibacterial, Antifungal and Ecotoxic Effects of Ammonium and Imidazolium Ionic Liquids Synthesized in Microwaves

**DOI:** 10.3390/molecules25215181

**Published:** 2020-11-06

**Authors:** Jana Fojtášková, Ivan Koutník, Martina Vráblová, Hana Sezimová, Milan Maxa, Lucie Obalová, Petr Pánek

**Affiliations:** 1 Institute of Environmental Technology, VSB-Technical University of Ostrava, 17. listopadu 15, 708 00 Ostrava, Czech Republic; jana.fojtaskova@vsb.cz (J.F.); ivan.koutnik@vsb.cz (I.K.); lucie.obalova@vsb.cz (L.O.); petr.panek@vsb.cz (P.P.); 2 Faculty of Materials Science and Technology, VSB-Technical University of Ostrava, 17. listopadu 15, 708 00 Ostrava, Czech Republic; 3 Department of Biology and Ecology, Faculty of Science, University of Ostrava, Chittussiho 10, 710 00 Ostrava, Czech Republic; hana.sezimova@osu.cz; 4 TECHEM CZ, Ltd., Ondříčkova 1300/48, 130 05 Praha 3, Czech Republic; maxa@techemcz.cz

**Keywords:** ionic liquid, microwave synthesis, imidazolium, quaternary ammonium salt, ecotoxicity

## Abstract

Ionic liquids are increasingly used for their superior properties. Four water-immiscible ionic liquids (butyltriethylammonium bis(trifluoromethylsulfonyl)imide, octyltriethylammonium bis(trifluoromethylsulfonyl)imide, dodecyltriethylammonium bis(trifluoromethylsulfonyl)imide, butyl-3-methylimidazolium bis(trifluoromethylsulfonyl)imide) and their water miscible precursors (bromides) were synthesized in a microwave reactor and by conventional heating. The best conditions for microwave-assisted synthesis concerning the yield and the purity of the product are proposed. The heating in the microwave reactor significantly shortened the reaction time. Biocide and ecotoxic effects of synthesized ionic liquids and their precursors were investigated. All tested compounds had at least a little effect on the growth or living of microorganisms (bacteria or mold). The precursor dodecyltriethylammonium bromide was found to be the strongest biocide, but posed a risk to the aquatic environment due to its relatively high EC_50_ value in the test with *Vibrio fischeri*. We assumed that apart from the alkyl chain length, the solubility in water, duration of action, or type of anion can influence the final biocide and ecotoxic effect.

## 1. Introduction

Ionic liquids (IL) are increasingly used for their superior properties. Their typical feature is a melting point below 100 °C. Such low melting points are mainly due to their composition. The combination of a larger asymmetric organic cation with smaller inorganic counterparts reduces the energy in the crystal lattice and hence the melting point of the resulting ionic liquid. Room temperature ionic liquids (RTILs) are of particular interest for practical applications. Thanks to this property, they can replace common solvents; they can dissolve organic, inorganic, organometallic, and polymeric substances. Uses of ionic liquids are usually determined from the melting point up to their decomposition temperature. The main advantage of ionic liquids includes the very low vapour pressure caused by their ionic character. Due to this, they are considered as green substitutes for volatile organic industrial solvents. This feature reduces the risk of exposure and solvent loss due to evaporation, thereby reducing air pollution [1].

The synthesis of RTILs is relatively simple. Besides the classical two-step or one-pot ways [2], advanced methods have been developed [3]. Methods can be classified according to whether the solvent is present or not. The classical two-step solvent-free path involves quaternization and metathesis [2]. Quaternization is a nucleophilic substitution of S_N_2 and relatively long reaction times (of the order of several days) are required to achieve high yields. The strength of the nucleophilic agent influences the rate of this reaction. As an advanced technology, microwave irradiation has been employed. The reduction of the reaction time in the preparation of N-alkylimidazolium salts in the microwave field has been described by Varma and Namboodiri [4], who achieved a 4 to 5-fold reduction in the time for the preparation of 1-butyl-3-methylimidazolium chloride and bromide. The second step–metathesis (ionic exchange)—is based on the different solubility of the reactants and products in the aqueous medium. The reaction runs at elevated temperature and requires a short reaction time [4].

Microwave syntheses of imidazolium and ammonium ionic liquids have been studied to a limited extent. Deetlefs and Seddon, Khadilkar and Rebeiro [5,6] described the preparation of 1-n-butyl-3-methylimidazolium bromide. Bhatt et al. [7] performed a microwave synthesis of dicationic ammonium salts. Dong et al. [2] briefly described the microwave-assisted synthesis of [N2224] [Br] and [N2224] [NTf2]. Physico-chemical properties of RTILs can be tailored by modifying their structure. Ionic liquids composed of bis(trifluoromethylsulfonyl) imide anion (NTf2) and different cations are expected to be useful for many practical applications in a wide range of areas, including electrochemistry, synthesis, and separation processes [8].

RTILs were often used in environmentally friendly applications, e.g., for aquatic pollutant extraction. AlSaleem et al. [9] studied the possibilities of extraction and oxidative destruction of halogenated carbohydrates by ionic liquids with bis(trifluoromethylsulfonyl) imide anion. Cacho et al. [10] tested the ability of extraction of organophosphate pesticides by [C4MIM][NTf2] from water with a yield greater than 97%. Yao et al. [11] extracted several drugs including paracetamol and ibuprofen from water with a yield greater than 91%. Cull et al. [12] achieved similar efficiency when erythromycin was extracted to [C4MIM][PF_6_] and butylacetate, whereas Manic et al. [13] extracted erythromycin to [C4C1pyrr][NTf2] with a yield of over 80%. The emulsion liquid membrane prepared from [C4MIM][NTf2] was tested for phenol, 2-chlorophenol, and 4-nitrophenol extraction from water [14]. Goyal et al. [15] stabilized a [N8881][Cl]-based emulsion liquid membrane by [C4MIM][NTf2] and extracted 97% of chromium from water. Kermanioryani et al. [16] used [C4MIM][NTf2] for the extraction of methylene blue from aqueous solution with an efficiency of 91.3%. Extraction of energetic materials-(2,4,6 trinitrotoluene), RDX (hexahydro-1,3,5-trinitro-1,3,5 triazine) and tetryl (2,4,6-trinitro-phenylmethylnitramine) from contaminated wastewater by a number of ionic liquids including [C4MIM][NTf2] was also studied [17]. Current status and future prospects of ionic liquid mediated technologies in wastewater treatment were reviewed recently [18].

Ionic liquids often exhibit an effect on living microorganisms [19,20]. Studies showing toxicity to bacteria [21,22] and fungi [23], and to the influence of enzymatic activity [24,25,26] have been published. Quaternary ammonium salts (QAS) are used as effective antiseptic agents under designation as cationic agents or cationic detergents [27]. In clinical practice, they are used for pre-operative disinfection of intact skin, application to mucous membranes, and disinfection of non-critical surfaces. The fact that QAS are membrane-active agents has been known for many years. Salton [28] proposed the following scheme of action of cationic agents on living microorganisms: (i) adsorption of the agent to the cell wall and its penetration into the cell wall; (ii) reaction with the cytoplasmic membrane (lipid or protein) followed by membrane disorganization; (iii) leakage of low molecular weight intracellular matter; (iv) protein and nucleic acid degradation; (v) wall decomposition by autolytic enzymes. The effect is facilitated by the interaction of the positive charge on the quaternary ammonium group with the polar groups contained in the phospholipid heads [29]. QAS is also believed to damage the outer membrane of gram-negative bacteria, thereby promoting their uptake. Moreover, QASs are sporostatic, they inhibit the growth of spores (the development of a vegetative cell from germination of spores) but not the actual germination processes (development from dormancy to a metabolically active state), although by an unknown mechanism. Similarly, QASs are not mycobactericidal, but the actual effects on mycobacteria have been studied by Russell [30].

Ionic liquids can be used as disinfectants against viruses. The effectiveness of the virus disinfectant is largely influenced by the type and structure of the virus. Non-lipid viruses generally need to be killed using broad-spectrum disinfectants. Sensitivity to lipophilic-type biocidal agents, e.g., 2-phenylphenol (cationic ionic liquid), chlorhexidine, and isopropanol, can be expected for viruses with a lipid envelope of hydrophobic nature [27]. Coronaviruses are enveloped (+)ssRNA viruses, with a lipid envelope. Rabenau et al. [31] tested the effectiveness of eight disinfectants against coronavirus SARS-CoV. Several selected formulations included two agents based on a 0.5% cationic ammonium salt mixture. Both benzalkonium chloride mixed with dodecylamine, and benzalkonium chloride mixed with glutaraldehyde and didecyldimethylammonium chloride, showed inactivation of SARS-CoV after 30 min. In their review, Kampf et al. [32] gathered information from studies on coronavirus’s resistance against biocidal agents. Wood and Payne [33] showed very good antiviral effects of benzalkonium chloride against human coronavirus ATCC VR-759. Similar conclusions were reached by Saknimit et al. [34] testing benzalkonium chloride against canine coronavirus strain I-71. Pratelli [35] confirmed the efficacy of benzalkonium chloride against canine coronavirus in strain S378 and found good antiviral effects of didecyldimethylammonium chloride in the same strain.

At present, it is constantly necessary to determine the possible toxic effects of ionic liquids on the natural ecosystem [20]. Flieger et al. [36] reviewed toxicity tests of a wide range of ionic liquids. Stolte et al. [37] tested the toxicity of a number of imidazolium salts. Cao et al., Erfurt et al. [38,39] studied cytotoxicity of selected ILs. However, the imidazolium and ammonium ILs presented in this work were not completely studied. One of the most widely used methods for assessing the toxicological risks of substances is ecotoxicological testing, which determines the concentration of a test substance that causes an effect/inhibition/mortality on the test organism. *Vibrio fischeri* bacteria are one of the most frequently studied organisms in the study of toxicological evaluation of ionic liquids [40,41,42]. *Vibrio fischeri* is commonly found in the environment, so this test is suitable for its screening and assessment of possible risks [43].

The aim of this work was to synthesize ammonium and imidazolium ionic liquids and their precursors in the microwave field and to test their biocide (antibacterial and antifungal) and ecotoxic effects. Microwave-assisted synthesis is believed to shorten the overall time and reduce energy consumption. Our purpose was to test the conditions under which the synthesis will be performed and compare the obtained results with the conventional heating method. With respect to physico-chemical properties of synthesized ionic liquids, we hypothesized the possible application of ILs as biocides, e.g., additives to paints and coatings (dispersants with antibacterial and antifungal properties), components in antibacterial cleaners, or disinfection agents in wastewater treatment. Simultaneously we evaluated possible ecotoxic effects especially for those substances that affect the growth of bacteria in the aquatic environment.

## 2. Materials and Methods

### 2.1. Materials

The following chemicals were used for the synthesis of ILs: 1-bromobutane (Sigma-Aldrich, 99%), 1-bromooctane (Acros Organics, 99%), 1-bromododecane (Acros Organics, 98%), triethylamine (Acros Organics, 99% pure), 1-methylimidazole, 1-chlorobutane (Sigma Aldrich, 99,5%), diethylether (Lach-Ner, 99%), *n*-pentane (Penta, p.a.). The 1-methylimidazole was purified by distillation and drying with potassium hydroxide. The other chemicals were used as received.

### 2.2. Synthesis of 1-Butyl-3-Methylimidazolium Bromide

A three-necked flask equipped with a magnetic stirring bar was charged with 1-methylimidazole (0.1 mol) and 1-bromobutane (0.11 mol) during synthesis under a nitrogen atmosphere. The synthesis was carried out by conventional and microwave heating. The mixture was heated to 80 °C under constant stirring. After completion of the reaction, the unreacted reactants were removed on a rotary evaporator under reduced pressure at a temperature up to 80 °C. Subsequently, the product was shaken with 5 × 25 mL of diethylether. The obtained IL was dried in a 1 mbar vacuum at 70–80 °C for 5–6 h.

### 2.3. Synthesis of 1-Alkyl-(Triethyl)Ammonium Bromide

A flat-bottomed flask equipped with a magnetic stirring bar was charged with triethylamine (0.64 mol) and 1-bromoalkanes (0.63 mol) with different alkyl chain lengths from C4 through C8 to C12. The synthesis was solvent-free and was carried out by conventional and microwave heating without using an inert atmosphere. The mixtures were heated from 100 to 140 °C under constant stirring. When the experiment finished, a biphasic mixture was formed (unreacted reaction mixture and 1-alkyl-(triethyl)ammonium bromide). 1-alkyl-(triethyl)ammonium bromide was in the form of a white insoluble precipitate. The precipitate was filtered and washed with 5 × 25 mL n-pentane to remove impurities. The obtained IL was dried in a 1 mbar vacuum at 70–80 °C for 5–6 h.

### 2.4. Ion Exchange (Metathesis)

The simple anion that had been synthesized in the first step (bromide) was then exchanged for the bulk anion of the lithium salt of bis[trifluormethyl(sulfonyl)]imide. To a flat-bottomed flask equipped with a magnetic stirring bar, equimolar amounts of ammonium salt and lithium salt were added. The mixture was dissolved in an adequate amount of water. The ion exchange was solvent-free and was carried out by conventional heating without using an inert atmosphere. The mixture was heated to 50 °C under constant stirring for 5–6 h. The ion exchange product was not soluble in water. The product was shaken with 150 mL diethyl ether and with 150 mL water. On a rotary evaporator under reduced pressure and at a temperature not exceeding 80 °C, the volatiles were evaporated together with water, until a constant weight was obtained.

### 2.5. Product Analysis

The content of water was determined by Karl Fischer titration using TitroLine 7500 KF titrator. The determination of dissociated bromides was done by argentometric titration using the automatic titrator T5 (Mettler-Toledo GmbH, Greifensee, Switzerland). The equivalence point was detected potentiometrically.

Liquid chromatography was used to determine the purity of imidazolium-based ionic liquids. Analysis was carried out using a liquid chromatograph equipped with model Nexera XR pumps and a SPD-M20A diode array detector (Shimadzu Corporation, Kyoto, Japan). The chromatographic separation was performed on a Phenomenex Kinetex Biphenyl analytical column (150 × 4.6 mm i.d., 2.6 µm) equipped with a guard column using an isokrat mode. Mobile phase was a mixture of 60% (70% methanol + 30% 0.054 M formic acid with ammonium acetate, pH 3.6) and 40% µH_2_O. The flow rate was 0.5 mL.min^−1^. The injection volume was 20 µL. The chromatographic system operated at 25 °C. For quantitative analyses selective detection was performed at 210 nm.

For ammonium-based ionic liquids the content of the main component was determined indirectly by determining by-products. GC-MS/FID analyses were carried out on a 7890A GC system equipped with a flame ionization detector and a single quad detector (Agilent Technologies, Inc., Santa Clara, CA, USA). A DB-5MS UI fused silica column (Agilent, 30 m × 0.32 mm ID, 0.5 µm film thickness) was used. The flow rate of the carrier gas (Helium) was set at 1.5 mL.min^−1^. The temperature program was as follows: 80 °C for 2 min, 30 °C.min^−1^ up to 250 °C, hold time 3 min. The injector temperature was 180 °C, in the split mode 20:1. The injection volume was 1 µL. The FID temperature was 250 °C.

Thermal gravimetric analysis (TGA) was performed with instrument LECO TGA 701, elemental composition C, H, N by LECO CHN 628 instrument (both from LECO Corporation, St. Joseph, MI, USA). The structure of products was confirmed by FTIR spectra, measured by Nicolet iS10 FTIR spectrometer with ATR accessories (Thermo Fisher Scientific, Waltham, MA, USA).

### 2.6. Biocide Tests

Biocide tests were performed within two experiments. In Experiment 1, the effects of ionic liquids on microorganisms compared to standard biocides were studied. The commercially available disinfection agent benzalkonium chloride (purity ≥95.0%, Sigma-Aldrich) was used as a standard. In Experiment 2, the half-maximal effective concentration (EC_50_) was determined for ILs.

Ionic liquids or standards were dissolved in demineralized water (Experiment 1) or a mixture of dimethyl sulfoxide (DMSO) and water (maximum of 10% *v*/*v*; Experiment 2) to the final amount of IL 2 wt.% in solution (Experiment 1) or concentration series 0.00 (negative control), 0.25, 0.74, 2.22, 6.67 and 20.00 g.L^−1^ (Experiment 2). Tested microorganisms were chosen based on their presence in the environment. In Experiment 1, *Penicillium candidum* from Camembert cheese, aerobic bacteria from wastewater treatment plants (WWTP; commercially available Bio-Enzym, Bioprospect, Czech Republic), and aerobic bacteria present in a digestate from a biogas reactor (operated at Institute of Environmental Technology, VŠB-TU Ostrava) were used. In Experiment 2, commercially available *Bacillus subtilis* (spore suspension 10^7^ CFU/mL, Merck) was tested. Samples with microorganisms were diluted in demineralized water (1 g per 100 mL) and activated for 30 min at 30 °C. The concentration of microorganisms and conditions of activation and cultivation, the same as the effect of DMSO on growth, were verified experimentally. Both solutions were pooled at a ratio of 1:1, therefore the concentration of microorganisms and ILs in the solution dropped to half of the previously mentioned values. Before testing, the contact time of microorganisms with ILs in solution was 1 h.

Biocide tests were performed on sterile total aerobic bacteria, yeasts and molds paddle testers (Hach Company, Loveland, CO, USA). Each tester contains two growth media; one for total aerobic bacteria, and a second for yeasts and molds. According to tested microorganisms, one of the media was chosen for testing. Solutions of ILs with microorganisms were put in drops (volume 3 μL) on the appropriate growth medium. Negative (solution of microorganisms in demineralized water) and positive (solution of microorganisms in water with standard biocide) controls were used in each tester. Testers were cultivated in a cultivation box tempered to 30 °C for 48 h. After cultivation, the size of microbial colonies was assessed from photographs in ImageJ software (Rasband, W.S., U. S. National Institutes of Health, Bethesda, MD, USA). The inhibitory effect (in %) was calculated as

(1 − *A*_(IL)_/*A*_(NC)_) (1)

where *A*_(IL)_ is the area of microorganism colony when treated with IL (in cm^2^) and *A*_(NC)_ is the area of microorganism colony in demineralized water (negative control) (Experiment 1). The dependency of inhibition effect on the concentration of IL in solution was expressed as half-maximal effective concentration. Data were evaluated using Origin Pro 2018 software. The equation of inhibition dose-response curve (2) was used for fitting [44] and (EC_50_) calculation (Experiment 2):

Y = Bottom + (Top-Bottom)/(1 + ((X^HillSlope)/(EC_50_^HillSlope))) (2)

where Bottom means basal response, Top means maximal response, and HillSlope is a gradient of the curve.

### 2.7. Ecotoxicity Tests

The luminescent bacteria test was performed according to ISO 11348 [45]. The bacteria *Vibrio fischeri* were obtained from Dr. Bruno Lange GmbH & Co. KG (Berlin, Germany) as liquid-dried cells (product LCK 480). The bacterial culture was cultivated according to the ISO 11348 procedures and was prepared fresh from the stock culture before each test.

The bacteria *Vibrio fischeri* were exposed to a dilution series of the studied samples and their light emission was determined after incubation. Additionally, negative and positive controls with potassium dichromate were tested. The light emission of the bacteria in the samples was measured after 15 and 30 min. The tests were performed at 15 °C and with two replicates. All measurements were performed using the LUMIStox300 luminometer (Hach-Lange GmbH, Düsseldorf, Germany).

Several dilutions for each of studied samples were prepared using distilled water (ILs Br) or DMSO (ILs NTf2), respectively. The different concentration ranges for these compounds were 0.08–25 mg.L^−1^ for [N222, 12][Br], 3.91–1000 mg.L^−1^ for [C4MIM][Br], 8–2500 mg.L^−1^ for [N2228][Br] and 2.4–62.5 mg.L^−1^ for [N2224][Br]. For ILs NTf2, the concentration range was 0.1–1 μL.mL^−1^ for [N222,12][NTf2], [N2228][NTf2] and 1–200 μL.mL^−1^ for [N224][NTf2], [C4MIM][NTf2].

## 3. Results and Discussion

### 3.1. Ionic Liquid Syntheses

The use of microwave heating in the synthesis of selected ILs resulted in a noticeable reduction in reaction times while achieving acceptable yields (Table 1) in accordance with results published previously [4]. Purity information for the synthesis of selected ILs in a microwave reactor is not available in the literature in sufficient quantity, so the results cannot be compared with other studies. Synthesis in the classical way is very well studied and there are many scientific articles on this topic. Physico-chemical parameters of synthesized ILs, including NMR spectra, can be found in the literature [46,47,48,49]. The yield of ammonium ionic liquids did not exceed 31% when conventional heating was applied up to 48 h, whereas in the microwave reactor, the yields between 58 and 86% were achieved in less than 12 h. The smallest effect of using microwaves was seen in the synthesis of [C4MIM][Br], which was strongly exothermic and required a very slight application of heating. The largest reduction in reaction time was achieved with [N222,12] [Br], where at the same time the yield almost doubled. The MW synthesis proceeded without the addition of a solvent. The addition of a solvent to the reaction mixture helps to increase the effect of microwave radiation and could significantly reduce the reaction time, but as a result may contribute to new problems in the purification of the final synthesis product. The low boiling point of butyl bromide causes a temperature limitation of the reactions and a reduction in yields. This disadvantage can be eliminated by using a high-pressure reactor.

A water content of 0.04–3.27 wt.% is considered the largest contaminant in synthesized ionic liquids. The residual water content of ILs is primarily related to the nature of the anion. The purification system was effective to achieve the highest purity of ionic liquids. All synthesized products were verified by infrared spectroscopy and elemental analysis. All quaternisation products are odourless white crystalline substances. Ion exchange products are very viscous colourless substances; [N2228] [NTf2] is mostly yellowish.

### 3.2. Biocide and Ecotoxic Effects

**Figure 1:**
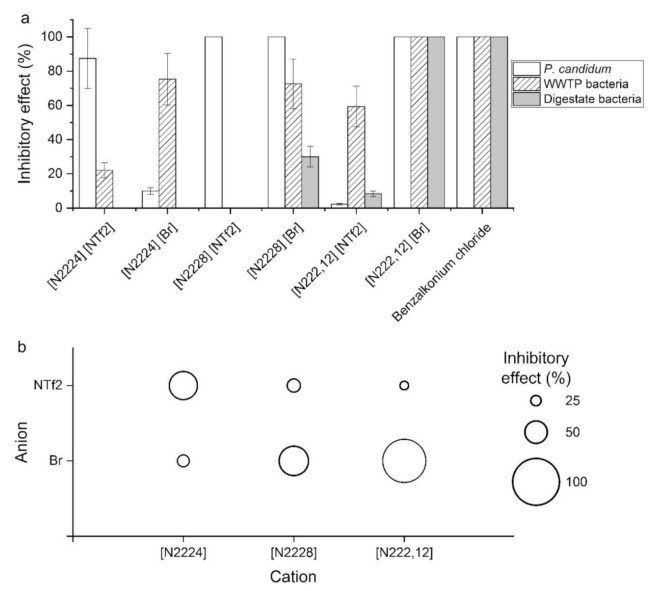
Inhibitory effect of ILs on three groups of microorganisms. (**a**) Relative inhibitory effect of QAS and their precursors, and standard inhibitor benzalkonium chloride, on the growth of *Penicilium candidum*, WWTP bacteria, and digestate bacteria. Bars represent standard deviations. (**b**) Visualization of inhibitory effects on all tested species (calculated as a sum of partial effects) concerning the anion and cation. ILs are aligned from left to right by extending the alkyl chain.

Inhibition of growth after contact with ILs in the aquatic environment was tested for three different species of microorganisms (Figure 1). Disinfection agent benzalkonium chloride inhibited all microorganisms in solution with a concentration of the inhibitor of 1 wt.% and 1 h of contact time. A similar inhibitory effect was observed for [N222,12][Br]. *Penicilium candidum* was highly inhibited by four from six tested ILs ([N222,12][Br], [N2228][Br], [N2228][NTf2] and [N2224][NTf2]). On the other hand, bacteria from the digestate were almost unaffected by all ILs except [N222,12][Br] and standard benzalkonium chloride. More than 60% inhibition of growth of WWTP bacteria were observed for all bromides and benzalkonium chloride. It can be summarized that bromides influenced microorganism growth more than triflimides (NTf2) and that triflimides were better fungicides than bactericides. The extending alkyl chain led to a higher inhibitory effect in bromides, but a lower inhibitory effect in triflimides. This fact is surprising and can be attributed to different solubility of ILs and their precursors in water. While bromides are very soluble in water (tens of wt. percent), the solubility of triflimides is very low (units of wt. percent). ILs containing shorter alkyl chains are more soluble than ILs with longer alkyl chains. Therefore, due to the high solubility of bromides, their biocide effect could be more affected by the length of the alkyl chain, whereas the biocide effect of triflimides could be closely related to water solubility. The dependence of inhibitory effect during the formation of biofilms on the length of the alkyl chain was previously shown for imidazolium ILs. Compounds (chlorides) with longer side chain lengths showed potent activity even at lower concentrations as compared to ILs with shorter chain lengths [50]. The ecotoxic effect of imidazolium ionic liquids with Cl^−^, PF_6_^−^ and BF_4_^−^ anions with longer chain lengths was also increased [51]. Longer alkyl chains were probably incorporated into the polar headgroups of the phospholipid bilayer, which led to membrane damage of microorganisms [52]. Contrary to these findings, biodegradation of phosphonium ionic liquids containing bromide was low and not dependent on alkyl chain length, and the presence of triflimide anion instead of bromide further inhibited the biodegradation [53].

**Figure 2:**
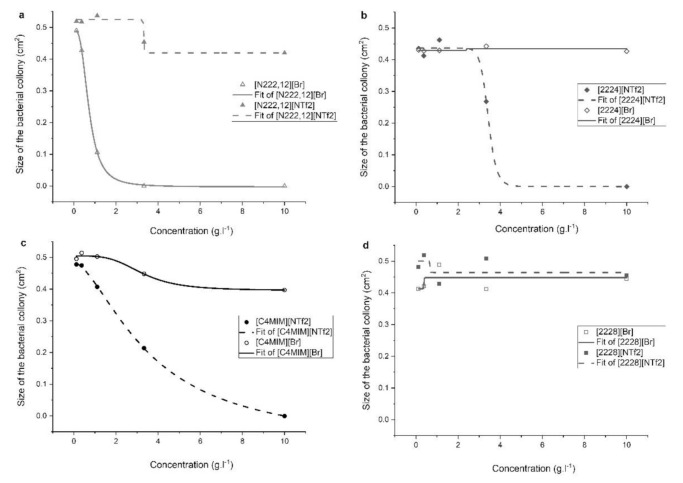
Concentration dependency of the inhibitory effect of ILs on the growth of *Bacillus subtilis*. Ammonium (**a**,**b**,**d**) and imidazolium (**c**) ionic liquids and their precursors were tested.

The effect of concentration of ILs in solution on the growth of microorganisms was tested for *Bacillus subtilis*. The concentration range 0–10 g.L^−1^ was set after preliminary results and concerning the low solubility of ILs in water. Only three from eight tested ILs ([N222,12][Br], [N2224][NTf2] and [C4MIM][NTf2]) exhibited a significant inhibition effect on the growth of *Bacillus subtilis* in this concentration range (Figure 2). Parameter EC_50_ was the highest for [N222,12][Br] (0.714 g.L^−1^), whereas [N2224][NTf2] and [C4MIM][NTf2] had EC_50_ values of 3.421 and 3.599 g.L^−1^, respectively. Other ILs exhibited a very low ([C4MIM][Br]) or negligible biocide effect. In this case, DMSO (10 wt.%) was used to improve the water solubility of studied compounds, therefore results obtained in this experiment may be slightly different from the previous experiment (Figure 2). Nevertheless, the effect of DMSO itself was tested independently, and no inhibition of growth of *B. subtilis* was found up to 15 wt.% solution of DMSO in water.

Similarly to Experiment 1, triflimides with shorter alkyl chain ([N2224][NTf2] and [C4MIM][NTf2]) and bromides with longer alkyl chain ([N222,12][Br]) have a higher potential to inhibit the growth of *B. subtilis*.

Acute toxicity in the aquatic environment was studied and EC_50_ values were obtained for *Vibrio fischeri* (Table 2). The samples of ionic liquids containing bromides showed different toxicological index EC_50_ values determined by the acute toxicity test on the luminescent bacteria *Vibrio fischeri*. The highest toxic effect was detected for [N222,12][Br] and a slightly smaller toxic effect for [N2224][Br]. This value is comparable to the EC_50_ for the reference substance, potassium dichromate (30 min EC_50_ = 2.8 mg.L^−1^). [C4MIM][Br] showed a significantly lower toxic effect, while [N2228][Br] exhibited a negligible toxic effect. In the case of ionic liquids containing the NTf2 group, the obtained EC_50_ values were quite similar for [N2228][NTf2] and [N222,12][NTf2], and in general, both chemicals can be considered as toxic for the environment using *Vibrio fischeri* as a bioindicator. Ecotoxicity of [N222,12][NTf2] was comparable to ecotoxicity of benzalkonium chloride (EC_50_ 0.5 mg.L^−1^ [54]). The highest EC_50_ value was 30 min EC_50_ 120.93 mg.L^−1^ for [N2224][NTf2]. [C4MIM][NTf2] was found to be moderately toxic in the aquatic environment (EC_50_ 18.91 mg.L^−1^ for *Daphnia magna*) [55], which is in agreement with our results.

According to Couling et al. [56], many of the imidazolium compounds were more toxic to *Vibrio fischeri* than common solvents such as acetonitrile, acetone, and methanol. On the contrary, low alkyl chain length quaternary ammonium ILs and those ILs containing choline as the cation were relatively nontoxic to *Vibrio fischeri* in their study. We found a similar trend, i.e., relatively low acute toxicity for [N2224][NTf2] compared to higher acute toxicity of [N2228][NTf2] and [N222,12][NTf2] with longer alkyl chains.

Couling et al. [56] assessed the factors responsible for ionic liquid toxicity to aquatic organisms via quantitative structure–property relationship modelling. Their results showed increasing alkyl chain length increases the IL toxicity for both *Vibrio fischeri* and *Daphnia magna*, and that cation properties have a larger effect on toxicity than anion properties. Nevertheless, bromides in our study usually showed a different ecotoxic effect on *Vibrio fisheri* than triflimides, which points to the non-negligible influence of the anion on acute toxicity. The inhibition of the growth of microorganisms in biocide tests was also species-dependent, with a balanced and strong inhibitory effect of [N222,12][Br] in all experiments. Similarly to our results, Sandbacka et al. [57] found relatively high acute toxicity (among 12 surfactants) of [N222,12][Br] to *Daphnia magna*. The exchange of [Br] to [NTf2] reduced the biocide effect, but not the acute ecotoxic effect. This could be influenced by the duration of action; in biocide tests, the contact time of compounds with microorganisms was 1 h in solution and another 48 h during testing, whereas acute toxicity was examined within 15 and 30 min. Therefore, we assume that the length of the alkyl chain of the cation is not the only feature that causes the inhibitory effect on microorganisms in the aquatic environment; the solubility in water, duration of action, type of anion, and possible other compound characteristics can influence the final biocide and ecotoxic effect.

## 4. Conclusions

The classical synthesis of tetraalkylammonium and imidazolium salts is a well-studied synthesis that leads to products of high purity. However, the reaction time for classical synthesis is on the order of tens of hours to days. Preparation of ionic liquids in a microwave reactor should contribute to reduction in reaction time of the synthesis, and lead to a higher purity of the product and a higher yield. Several new findings were identified in the presented work. (i) The microwave field has been shown to have a beneficial effect on the synthesis of both imidazole and ammonium ionic liquids. We defined the conditions for microwave-assisted synthesis that led to a sufficient yield and purity of products. (ii) All tested compounds had at least a little effect on the growth or living of microorganisms (bacteria and mold). We showed that results of biocide tests depended on tested species and were influenced not only by the length of the alkyl chain of IL, but also on its solubility in water and type of anion. In general, bromides influenced microorganism growth more than triflimides (NTf2), and triflimides were better fungicides than bactericides. Dodecyltriethylammonium bromide showed strong biocide and ecotoxic effects in all tests. (iii) The antibacterial and antifungal effects of studied ILs together with an ecotoxicity that was comparable or lower than the widely used benzalkonium chloride predestine these compounds for testing in applications in the field of paints and coatings, antibacterial cleaners or, in the case of low water-soluble ILs, as disinfection agents for wastewater treatment.

## Figures and Tables

**Table 1:** Results of ionic liquid syntheses.

Anion		[Br]					[Ntf2]		
**Reaction**		Conventional		Microwave			Metathesis		
**Cation**	Purity (wt.%)	Yield (wt.%)	Time (min)	Purity (wt.%)	Yield (wt.%)	Time (min)	Purity (wt.%)	Yield (wt.%)	Time (min)
**[C4MIM]**	98.3	62.9	240	99.6	74.8	180	97.7	56.3	1200
**[N2224]**	95.4	21.9	1400	99.7	85.6	560	96.0	57.9	300
**[N2228]**	97.5	26.8	1440	99.1	63.4	700	96.5	53.6	360
**[N222,12]**	99.8	30.2	2880	98.2	58.2	480	99.2	57.0	300

**Table 2:** Effective concentrations and their corresponding standard deviations obtained in *Vibrio fischeri* test.

Ionic Liquid	15 min EC_50_ (mg.L^−1^)	30 min EC_50_ (mg.L^−1^)
[C4MIM][Br]	375.96 ± 17.78	341.66 ± 19.05
[N2224][Br]	2.70 ± 0.28	2.99 ± 0.43
[N2228][Br]	1588.32 ± 28.06	1179.51 ± 34.97
[N222,12][Br]	0.40 ± 0.04	0.37 ± 0.07
[C4MIM][NTf2]	18.47 ± 0.31	14.97 ± 1.36
[N2224][NTf2]	142.33 ± 18.92	120.93 ± 17.87
[N2228][NTf2]	0.27 ± 0.04	0.31 ± 0.03
[N222,12][NTf2]	0.51 ± 0.03	0.45 ± 0.01
